# Alerting network, cognitive flexibility in children with attention deficit hyperactivity disorder and the moderating effect of neuroticism

**DOI:** 10.1097/MD.0000000000035583

**Published:** 2023-10-13

**Authors:** Xiang Zhang, Shaoxia Wang, Qianyun Liu, Chujun Wu, Yunyun Du, Yanrong Wang, Jianqun Fang

**Affiliations:** a Department of Psychiatry, Ningxia Medical University, Ningxia, China; b Mental Health Centre, General Hospital of Ningxia Medical University, Ningxia, China.

**Keywords:** alerting, attention deficit hyperactivity disorder, attention network, cognitive flexibility, neuroticism

## Abstract

Attention deficit hyperactivity disorder (ADHD) is a prevalent neurodevelopmental disorder, and cognitive flexibility is a sub-component of executive functioning. Studies have shown impairments in cognitive flexibility in ADHD, which is affected by attentional processes. Personality, as a long-standing trait, has a profound effect on ADHD. However, previous studies have not assessed the relationship between attentional function, personality traits, and cognitive flexibility in children with ADHD. This study explored the association between attention networks, personality, and cognitive flexibility in ADHD, filling a gap in the related field. We expect our findings will provide insights into and clues for the prevention and interventional treatment of ADHD. This study primarily aimed to analyze differences in cognitive flexibility between individuals with ADHD and those without and further examine associations between attention networks, personality, and cognitive flexibility in children with ADHD. Overall, 55 children aged 7 to 11 years diagnosed with ADHD and 40 children without ADHD participated in this study. Cognitive flexibility, personality traits, and attentional networks were assessed using the Wisconsin Card Sorting Test, Eysenck Personality Questionnaire, and Attention Network Test, respectively. Additionally, the association between personality traits and strong attentional functioning and cognitive flexibility was investigated using multiple regression analysis. Children with ADHD had significant deficits in cognitive flexibility. A multiple regression analysis revealed that the alerting effect was highly associated with cognitive flexibility at high levels of neuroticism. This association was not salient in individuals with low levels of neuroticism. This study demonstrated that the ADHD group experienced lower cognitive flexibility than the control group. In addition, we showed the effect of neuroticism and alerting networks on cognitive flexibility. These findings may help psychiatrists provide intervention strategies to mitigate the impairment of social functioning in ADHD with cognitive spirituality deficits.

## 1. Introduction

Attention deficit hyperactivity disorder (ADHD) is a prevalent neurodevelopmental disorder characterized by persistent inattention and hyperactive or impulsive behavior that impairs children daily functioning.^[1]^ ADHD frequently occurs in school-aged children and may be comorbid with oppositional defiant disorder and mood disorders, placing a severe burden on the affected family, life, school, and social life.^[2]^ A recent global meta-analysis showed a prevalence of 3.4% in children and adolescents.^[3]^ A Meta-analysis showed that the prevalence estimate of 5.3% of ADHD among children and adolescents in China was 6.26%.^[4]^

Impaired mental health and social functioning in people with ADHD are intimately associated with abnormal alterations in cognitive flexibility.^[5,6]^ Cognitive flexibility, the ability to transition between tasks or goals, allowing individuals to adjust their thoughts or cognitive behavior appropriately in response to changes in the environment, is considered one of the critical sub-components of executive functioning (EF).^[7,8]^ In 1993, Eslinger and Grattan classified cognitive flexibility into 2 types: reactive and spontaneous. “Reactive” flexibility is the ability to change cognitive responses in reaction to changes in context. In contrast, “spontaneous” flexibility is the continuous flow of spontaneously sought ideas in response to open-ended questions.^[9]^

Although previous studies have explored cognitive flexibility in ADHD, their findings are contradictory.^[10]^ Many researchers have found significant impairments in cognitive flexibility in ADHD.^[10–18]^ Wixted, Sue, Dube, Potter^[10]^ used functional magnetic resonance imaging and found that the anterior cingulate gyrus, superior frontal gyrus, and inferior frontal gyrus, associated with cognitive flexibility, are less active in ADHD.

However, other studies have not identified impaired cognitive flexibility in ADHD.^[19–21]^ Rommelse, Altink, de Sonneville, Buschgens, Buitelaar, Oosterlaan, Sergeant^[19]^ showed that children with ADHD and their unaffected siblings did not exhibit cognitive flexibility deficits. The authors suggested that deficits in EF in ADHD may be due to deficits in cognitive functions, including attention, rather than directly affecting cognitive flexibility.^[19]^ Irwin, Kofler, Soto, Groves^[21]^ compared children with ADHD aged 8 to 13 years with controls and observed no significant evidence of a unique cognitive flexibility deficit. This finding suggests that cognitive flexibility in ADHD requires further investigation.

The primary mechanism underlying abnormal cognitive flexibility is an attention deficiency, which prevents access to accurate and continuous processing, memory, and eventual recall of information.^[15,22]^ A longitudinal study of typically developing children found that children with more advanced cognitive flexibility skills exhibited lower levels of hyperactivity/inattention behavior.^[18]^ For those diagnosed with ADHD, attention deficits are one of the most common symptoms of ADHD; to date, this disorder remains not undefined in terms of specific cognitive deficits.^[23]^ It is unclear which specific attentional ability is reduced in ADHD.^[24]^

According to Posner and Petersen model,^[25]^ attention comprises 3 mutually independent brain networks, each representing an attentional process. The alerting network is responsible for achieving and maintaining alertness. In contrast, the orienting network selects information from the environment by focusing on a particular input pathway, and the conflict network resolves the conflict that arises between competing stimuli.^[26]^ These networks are believed to be anatomically and functionally well separated.^[23]^ Alertness depends on the frontal, parietal, and thalamic areas and is affected by the noradrenergic system.^[26]^ The orienting network depends on the parietal and midbrain circuits and the pulvinar, and the cholinergic system plays a crucial role.^[25,27]^ The conflict network is considered to be associated with the dorsal anterior cingulate and the lateral prefrontal cortex, which are also dopaminergic systems.^[26]^ Fan, McCandliss, Sommer, Raz, Posner,^[28]^ to independently measure the efficiency of these 3 networks through a single task, invented the attention network test (ANT), which allows the efficiency of each attention system to be simultaneously assessed. This task showed not only the specific attention-impaired system in ADHD but also the apparent correlation between behavioral measures and the neural network of attentional processes. Consequently, the ANT has been well-received in research on attention disorders in ADHD. Most studies on children with ADHD only show differences between the alerting and conflict networks compared with the control group.^[23]^ However, there are hardly any historical papers on the relationship between the attention network system and cognitive flexibility in ADHD. Therefore, the present study will continue to explore the relationship between attentional networks and cognitive flexibility in ADHD.

Personality traits are closely associated with ADHD. Eysenck, Eysenck^[29]^ proposed a 3-factor model of personality, classifying personality as psychoticism, neuroticism, and extraversion. Additionally, they developed the Eysenck personality questionnaire (EPQ)^[30]^ based on this model, which has been widely used in personality assessments. In studies using the EPQ, high psychoticism and neuroticism scores are strongly associated with the manifestation of ADHD symptoms.^[31]^ This association has also been found in adult patients with ADHD and adolescent patients with ADHD.^[32]^ The interaction between extraversion and ADHD has been inconsistently reported in previous reports.^[33–35]^ In the study by Yin, Wu, Yu, Liu,^[36]^ personality factors were considered a moderating factor that moderates the effects of traumatic experience on PTSD among 457 survivors who experienced the Wenchuan earthquake. Neuroticism increases arousal symptoms of PTSD. Individuals with high neuroticism in the face of earthquake trauma are more likely to view the earthquake experience as a more negative and threatening event, and are more prone to maladaptive coping styles, and inflexible cognitions. Therefore, in the present study we presumed that personality factors may also moderate the relationship between attention and cognitive flexibility in ADHD.

Neuroticism is a personality trait characteristic of a tendency to experience negative emotions more frequently, intensely, more readily, and for more extended periods. One condition that individuals often confront is the necessity to dynamically adjust their actions and thoughts to respond to the changing demands of the environment. This means that individuals must maintain their purposes and goals over time while being flexible enough to shift from one thought or behavior to another when significant changes occur.^[37]^ Research in normal populations suggests that neuroticism, emotional disorders,^[38]^ and emotion dysregulation^[39–41]^ are related. People with high neuroticism scores were less willing to modify their behavior in response to feedback.^[42]^ Kashdan, Rottenberg^[43]^ suggest that people high in neuroticism experience excessive negative emotions, are tightly wrapped up in negative thoughts and behaviors, and have difficulty tolerating them, compromising their cognitive flexibility. In counterpoint, positive emotion states facilitate increased cognitive flexibility.^[43]^ One hundred seventy-four undergraduates tested on the modified version of the Continuous Performance Test (AX-CPT) under positive, neutral, and negative affect. The results found that the participants performed better cognitive flexibility under the mildly positive affect.^[37]^ It has been reported that HPA axis activity is implicated in negative clinical outcomes, irrespective of the presence or absence of psychiatric conditions.^[44]^ The aberrations in HPA axis function, primarily marked by heightened HPA axis activity, appear to exert a significant modulatory influence on adverse clinical outcomes, while deficiencies in stress response mechanisms contribute significantly to the manifestation of neuroticism and risk of suicide. Furthermore, a study has demonstrated that individuals with ADHD and comorbid psychiatric conditions may have an even higher risk of developing treatment resistance. Moreover, elevated concentrations of inflammatory mediators in both the peripheral blood and brain may also contribute to treatment resistance.^[45]^

Nevertheless, the above association remains further confirmed in the ADHD population. Therefore, personality factors, as persistent long-term characteristics, probably influence cognitive flexibility in children with ADHD. Based on these findings, we assume that neuroticism might be a risk factor for cognitive flexibility in children with ADHD. Moreover, the attentional performance of children with ADHD should also be taken into account. Although the attentional process is related to cognitive flexibility, the exact mechanism by which it currently affects cognitive flexibility is unclear, and its intertwined effect on personality has rarely been explored.

As mentioned earlier, a close correlation exists between personality traits, attention networks, and cognitive flexibility in ADHD. This study aimed to investigate whether children with ADHD have cognitive flexibility impairments and investigate the association between their attention networks and cognitive flexibility. Furthermore, we examined whether personality traits moderated these correlations. Additionally, we used the Wisconsin card sorting test (WCST) to measure cognitive flexibility, ANT to assess attention networks, and EPQ to measure personality dimensions and make the following hypotheses: the ADHD group showed lower cognitive flexibility compared to controls, decreased attention network effect and high neuroticism may predict cognitive flexibility, and personality traits may moderate the relationship between attention and cognitive flexibility.

## 2. Materials and methods

### 2.1. Participants

Overall, 95 children aged 7 to 11 years were included in this study. There were 55 (11 girls, 44 boys) and 40 children (20 girls, 20 boys) included in the ADHD and the control groups, respectively. Participants were excluded if they had an intellectual disability, autism spectrum disorder, other severe developmental disorders, schizophrenia, bipolar disorder, and other severe mental illnesses, or a Full-Scale IQ lower than 70. The children in the ADHD group were recruited from referrals to Psychiatric Clinics and Pediatric Clinics at the General Hospital of Ningxia Medical University, who visited the outpatient clinics and were diagnosed with ADHD according to the Diagnostic and Statistical Manual of Mental Disorders, Fifth Edition diagnostic criteria (DSM-V).^[1]^ All potential non-ADHD were recruited through advertisements in the Ningxia area and local public primary schools.

The diagnosis of ADHD was based on multiple aspects, including an interview with a trained psychiatrist; Face-to-face clinical observation of participants performance by psychiatrists; the participants medical history provided by parents; the severity of ADHD symptoms were assessed using the Swanson, Nolan, and Pelham, Version IV- Scale (SNAP-IV) - Chinese version.^[46]^ Of the participants, 10 were diagnosed with ADHD-H, 22 with ADHD-I and 23 with ADHD-C. Participants in the control group did not meet the current diagnostic criteria. The local institutional review board approved this study and written informed consent was obtained from all the children and their guardians before the assessment.

### 2.2. Measures

#### 2.2.1. ADHD severity.

The clinical severity of ADHD symptoms was assessed using SNAP-IV - Chinese version. The Chinese version SNAP-IV scale is commonly used in the clinical diagnosis of ADHD^[46]^ with good reliability and construct validity.^[47]^ It includes 3 subscales: inattention, hyperactivity/impulsivity, and oppositional defiant disorder. This scale symptoms are rated as a 4-point Likert scale (from 0 = not at all to 3 = very much).

#### 2.2.2. Wisconsin card sorting test.

This study measured cognitive flexibility using a computerized version of the WCST-128. There were 3 indicators of cognitive flexibility: number of categories completed (CC), perseverative responses (PR), and perseverative errors (PRE).^[48]^ Raw scores for all indicators are recorded. All indicators were calculated using the method described by the retest reliability (generalization coefficients) of WCST ranges from 0.37 to 0.72.^[49]^

We implemented Heaton, Chelune, Talley, Kay, Curtiss^[50]^ administration and scoring in the present study. Perseverative responses - when the dimension the participant is sorting by matches the perseverated-to-principle, the participant continually responds using an incorrect dimension even after receiving feedback that this response is incorrect. PRE - the wrong part of the PR. The Number of Categories Completed - The ten consecutive correct matching sequences completed by the participants in the test. The score ranges from 0 to 6.

#### 2.2.3. Eysenck personality questionnaire.

The EPQ was developed by Eysenck, Eysenck,^[30]^ and it is a self-report measure comprising 88 items on 4 subscales to assess personality traits. The EPQ includes 3 personality scores (psychoticism [P], extraversion [E], and neuroticism [N]) and a Lie (L) score. The Chinese version of the EPQ was revised by Gong Yaoxian and has been used for at least 40 years, with good reliability and validity.^[51]^

#### 2.2.4. Wechsler intelligence scale for children-IV-Chinese.

The WISC-IV-Chinese is commonly used to assess the intelligence of children and adolescents,^[52]^ and it comprises ten core subtests that are combined to form 4 metric scores. All of these were aggregated into one full-scale intelligence quotient, ranging from a minimum of 40 points to a maximum of 160 points. The split-half reliability of each subtest of the scale ranged from 0.78 to 0.92, and the split-half reliability of each composite score ranged from 0.87 to 0.97. The retest reliability of each subtest ranged from 0.71 to 0.86, and the reliability of the composite scores were above 0.8.

#### 2.2.5. Attention network test.

Considering the developmental characteristics of children attention, a researcher designed a child version of the ANT based on the adult version.^[53]^ The children version replaced the ANT background with blue and yellow fishes instead of the original black arrows. In the task, participants were required to identify the direction of the target arrow in a disturbance, with arrows pointing in different directions on either side.

**Figure 1.**
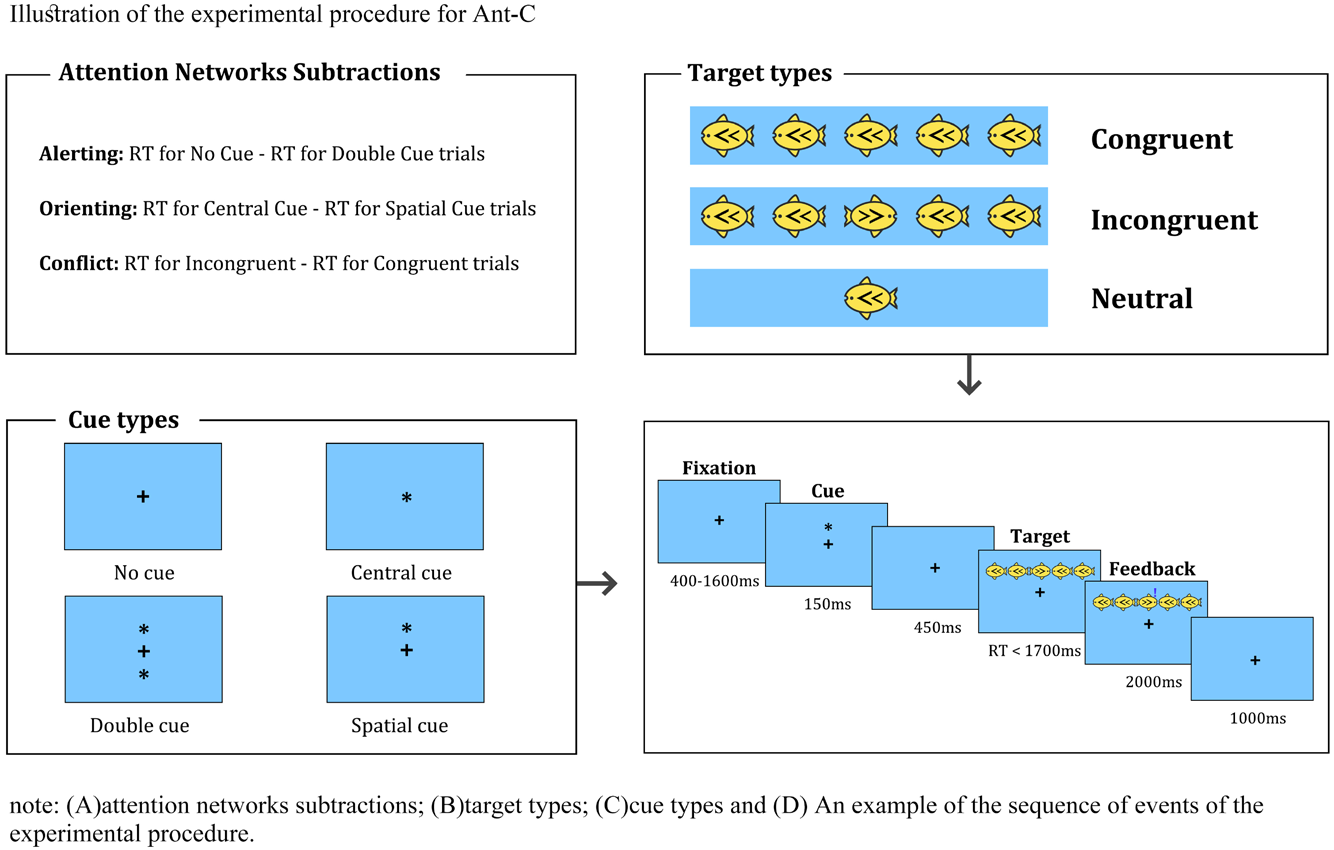
Illustration of the experimental procedure for child version of the ANT (ANT-C). (A) Attention networks subtractions; (B) target types; (C) cue types, and (D) an example of the sequence of events of the experimental procedure. RT = reaction time.

The child version of the ANT task was performed using E-prime software on computers. Figure 1 shows the experimental procedure for ANT. All stimuli were presented on a 19 inches computer screen, and each trial started with a central fixation cross, and the cue appeared on the screen after a random interval of 400 to 1600 ms. A cue (asterisk) appeared for 150 ms and might have appeared in various forms. A fixation cross would then appear in the center of the screen and last 450 ms. The target fish or flanking fish would appear on the screen immediately afterward and disappear after giving a reaction or lasting a maximum of 1700 ms. Finally, after another 1000 ms fixation period, a new trial started. The cueing conditions included the following: no cue fixation cross only, center cue -a “*” symbol located at the center of the screen, double cue-two “*” symbols each located above and below the fixation cross along the vertical meridian, and spatial cue -a “*” symbol located either above or below the fixation cross.

The ANT in this study comprised 24 practice trials and 3 experimental blocks of 48 trials each. Practice trials were not factored in the overall score since they were intended to familiarize participants with the rules. Although the practice trials provided feedback after each response, there was no feedback in the experimental area. Each trial represented 1 of the 12 conditions in equal proportions: 3 target types (congruent, incongruent, and neutral) × four cues (no cue, central cue, double, cue, and spatial cue). Additionally, the flanking conditions could be neutral (the central fish appeared alone), congruent (flanking fish pointed in the same direction), or incongruent (the flankers pointed in the opposite direction from the central fish). The participants sat at a distance of approximately 55 cm from the screen for the experiment. The participants needed to judge the direction of the centermost fish according to the instruction, pressing the “F” key when the fish was facing left and the “J” key when the fish was facing right. The response time (RT) and accuracy of each experiment trial were recorded.

The 3 attention network effects were manipulated as the mean RT difference between the task-specific conditions. Alerting network effect = mean RTno cue - mean RTdouble cue; orienting network effect = mean RTcentral cue- mean RTspatial cue; and conflict network effect = mean RTincongruent - mean RTincongruent. Trials with reaction times below 200 ms and those with incorrect responses were excluded.

### 2.3. Procedure

After agreeing to participate in this study, all participants parents or legal guardians signed an informed consent form, and a clinical interview was conducted to consolidate the clinical diagnosis. The assessment protocol included demographic information, attention network measures, personality traits, and cognitive flexibility. Additionally, all the participants completed the assessment process and received a written report that included their results afterward.

### 2.4. Statistical analyses

Descriptive statistics are the mean ± standard deviation (SD) or frequency (percentage). The Kolmogorov–Smirnov test was used to assess the normality of the variables. The ADHD and control groups were compared using the *t* test and χ^2^ test, respectively.

As the influence of multiple factors on cognitive flexibility was examined in the current study, we used a 2-step statistical analysis to investigate the correlation of the attention network system with personality and cognitive flexibility. In the first step, Pearson correlations was used to select possible predictors of cognitive flexibility for further analysis, including age, attention network effects, and personalities. Additionally, the multiple regression analysis was performed to assess the correlation between attention network, personality traits, and cognitive flexibility by controlling for the effects of other factors. Factors that are significant in the first step will be entered into the second step of multiple regression analysis.

According to the criteria proposed by Baron, Kenny,^[54]^ moderating effect was examined. In the present study, if attention network effect and hypothesized moderators (personality traits) were significantly associated with cognitive flexibility, then the interactions (attention network effects × hypothesized moderators) were further selected for multiple regression analysis to examine the moderating effects. Data analyses were performed using the SPSS software (version 22.0; IBM Corp., Armonk, NY). Simple slope analyses were conducted using Process v3.3. Two-tailed *P* values of < .05 were considered statistically significant.

## 3. Results

### 3.1. Descriptive statistics

Table 1 presents participants sociodemographic and EPQ scores, cognitive flexibility, and attention network effects. The ADHD group (n = 55) comprised 44 males and 11 females with a mean age of (8.84 ± 1.10) years, and the control group (n = 40) included 20 males and 20 females with a mean age of (8.98 ± 1.00) years. Differences between the 2 groups regarding sex (χ^2^ = 9.481, *P* = .004), with significantly more boys than girls in the ADHD group than in the healthy group. There were no differences in age (t = −0.629, *P* = .531), grade (t = −0.347, *P* = .729), full-scale intelligence quotient (t = −0.629, *P* = .531), EPQ-P scores (t = −1.863, *P* = .066), EPQ-E scores (t = 1.203, *P* = .232). Inattention (t = 12.256, *P* < .001), hyperactivity/impulsivity (t = 9.907, *P* < .001), and oppositional (t = 5.150, *P* < .001) on SNAP-IV scale were significantly different. The alerting effect (t = −4.335, *P* < .001), EPQ-N scores (t = 2.036, *P* = .035), PR (t = 3.164, *P* = .002), PRE (t = 3.438, *P* = .001), and CC (t = −3.353, *P* = .001) were significantly different between the 2 groups; children with ADHD had a significantly higher PR and PRE, and a significantly lower CC and alerting network effect than the controls.

**Table 1:** ADHD and control group differences in sociodemographic, personality, attention network effects and cognitive flexibility.

	ADHD group (n = 55)	Control group (n = 40)	χ^2^/t	*P*
Age (yr)	8.84 ± 1.10	8.98 ± 1.00	−0.629	.531
Sex			9.481	.004
Girls	11 (11.6%)	20 (21.1%)		
Boys	44 (46.3%)	20 (21.1%)		
Grade	2.91 ± 0.84	2.98 ± 1.00	−0.347	.729
FSIQ	102.38 ± 14.31	106.78 ± 8.43	−1.863	.066
SNAP-IV				
IA	18.51 ± 4.71	7.40 ± 3.82	12.256	<.001
H/I	13.87 ± 6.16	5.40 ± 2.67	9.097	<.001
Oppositional	9.15 ± 4.92	5.43 ± 1.81	5.150	<.001
EPQ				
P	4.07 ± 2.27	4.28 ± 3.03	−0.356	.723
E	16.25 ± 4.66	15.08 ± 4.8	1.203	.232
N	10.15 ± 4.61	8.40 ± 3.34	2.036	.035
ANT (ms)				
Alerting	26.44 ± 36.95	59.23 ± 35.61	−4.335	<.001
Orienting	49.78 ± 55.01	69.17 ± 43.88	−1.909	.059
Conflict	78.84 ± 62.68	75.64 ± 65.10	0.241	.810
WCST				
PR	37.53 ± 16.85	26.55 ± 16.48	3.164	.002
PRE	34.98 ± 16.57	22.83 ± 14.98	3.674	<.001
CC	3.38 ± 1.69	4.58 ± 1.74	−3.353	.001

ADHD = attention deficit hyperactivity disorder, ANT *=* attention network test, CC *=* number of categories completed, EPQ-E *=* Eysenck personality questionnaires-extraversion, EPQ-N *=* Eysenck personality questionnaires-neuroticism, EPQ-P *=* Eysenck personality questionnaires-psychoticism, FSIQ *=* full-scale intelligence quotient, H/I = hyperactivity/impulsivity, IA *=* inattention, PR *=* perseverative responses, PRE *=* perseverative errors, SNAP-IV *=* Swanson, Nolan, and Pelham, Version IV Scale, WCST *=* Wisconsin card sorting test.

### 3.2. Correlation analysis of personality, attention network effect, and cognitive flexibility

Table 2 shows the correlations for age, personalities, attention network effects, and cognitive flexibility using Pearson correlation analysis. Concerning attention network effect outcomes as measured by the ANT, only the alerting effect was negatively correlated with PR (r = −0.338, *P* < .05), PRE (r = −0.373, *P* < .01), and positively correlated with CC (*R* = 0.272, *P* < .05). The correlations between orienting and conflict effect with PR, PRE and CC were insignificant. Additionally, no significant relationship was observed between personality traits and cognitive flexibility. The conflict and orienting networks in the attention network were not significantly correlated with cognitive flexibility.

**Table 2:** Correlation of sociodemographic, personality, attention network effects, cognitive flexibility: Pearson correlation.

	PR	PRE	CC
Age	−0.114	−0.102	0.223
EPQ			
EPQ-N	0.229	0.258	−0.204
EPQ-P	0.019	0.032	−0.046
EPQ-E	−0.071	−0.033	0.013
ANT			
Alerting	−.338^*^	−.354^**^	.272^*^
Orienting	−0.037	−0.081	0.128
Conflict	0.054	0.01	−0.115

ANT *=* attention network test, CC *=* number of categories completed, EPQ *=* Eysenck personality questionnaire, EPQ-E *=* Eysenck personality questionnaires-extraversion, EPQ-N *=* Eysenck personality questionnaires-neuroticism, EPQ-P *=* Eysenck personality questionnaires-psychoticism, PR *=* perseverative responses, PRE *=* perseverative errors.

* *P* < .05, **P < .01.

### 3.3. Analysis of moderating effect

Following the description in Statistical Analyses, the significant variable in the first step entered the multiple regression analysis to examine the independent factors related to cognitive flexibility. After variables including sex, age, EPQ-P, EPQ-E, Orienting, conflict, and ADHD symptoms were controlled, Table 3 shows that EPQ-N was positively associated with PR (β = 0.305, *P* < .05) and PRE (β = 0.320, *P* < .05), and negatively associated with CC (β = −0.327, *P* < .05). Alerting effect was significantly associated with PR (β = −0.374, *P* < .05) and PRE (β = −0.367, *P* < .05), and positively associated with CC (β = 0.306, *P* < .05).

**Table 3:** Multiple logistic regression using for predicting cognitive flexibility in attention deficit hyperactivity disorder (ADHD) group.

	PR			PRE			CC		
	B	95% CI	β	B	95% CI	β	B	95% CI	β
Sex	9.358	(−4.514, 23.23)	0.224	7.114	(−6.455, 20.683)	0.173	−1.350	(−2.665, −0.035)	−0.322
Age	−0.793	(−5.626, 4.039)	−0.052	−2.063	(−6.79, 2.663)	−0.137	0.242	(−0.216, 0.7)	0.157
SNAP-IV									
IA	0.451	(−0.652, 1.554)	0.126	0.558	(−0.521, 1.637)	0.159	0.029	(−0.076, 0.133)	0.080
H/I	0.567	(−0.306, 1.441)	0.207	0.551	(−0.303, 1.405)	0.205	−0.084	(−0.166, −0.001)	−0.304
EPQ-P	0.299	(−2.006, 2.604)	0.040	0.372	(−1.882, 2.627)	0.051	−0.034	(−0.253, 0.184)	−0.046
EPQ-E	−0.386	(−1.481, 0.709)	−0.107	−0.270	(−1.341, 0.801)	−0.076	0.008	(−0.096, 0.112)	0.022
Orienting	−0.008	(−0.1, 0.085)	−0.025	−0.028	(−0.119, 0.062)	−0.094	0.004	(−0.005, 0.012)	0.115
Conflict	−0.011	(−0.094, 0.072)	−0.042	−0.028	(−0.109, 0.053)	−0.105	−0.001	(−0.008, 0.008)	−0.007
EPQ-N	1.115^*^	(0.101, 2.128)	0.305	1.151^*^	(0.164, 2.138)	0.320	−0.120^*^	(−0.216, −0.024)	−0.327
Alerting	−0.171^*^	(−0.303, −0.038)	−0.374	−0.165^*^	(−0.294, −0.036)	−0.367	0.014^*^	(0.001, 0.027)	0.306

Alerting× EPQ-N *=* interaction between alerting effect and neuroticism, CC *=* number of categories completed, CI *=* confidence interval, EPQ-E *=* Eysenck personality questionnaires-extraversion, EPQ-N *=* Eysenck personality questionnaires-neuroticism, EPQ-P *=* Eysenck personality questionnaires-psychoticism, H/I = hyperactivity/impulsivity, IA *=* inattention, PR *=* perseverative responses, PRE *=* perseverative errors, SNAP-IV *=* Swanson, Nolan, and Pelham, Version IV Scale.

* *P*< .05, ^**^P < .01.

Because EPQ-N and alerting effect were significantly associated with cognitive flexibility, the interaction terms of predictors and moderators (Alerting × EPQ-N) according to the criteria proposed by Baron, Kenny^[54]^ were also entered into multiple regression analysis.

Table 4 to 6 showed that the interaction between the EPQ-N and alerting was significant. Moreover, the interaction between alerting and EPQ-N was negatively correlated with PR (β = − 0.296, t = −2.222, *P* = .032; Table 4) and PRE (β = −.357, t = −2.786, *P* = .008; Table 5), but CC (β = 0.163, t = 1.247, *P* = .219; Table 6) was not significant, which suggested that neuroticism modulates the contribution of alerting effects to cognitive flexibility.

**Table 4:** Associated factors and moderators of cognitive flexibility perseverative responses (PR).

	Model I			Model II			Model III		
	β	t	*P*	β	t	*P*	β	t	*P*
Sex	0.224	1.358	.181	0.206	1.363	.180	0.162	1.107	.274
Age	−0.052	−0.330	.743	−0.092	−0.639	.526	−0.056	−0.407	.686
SNAP-IV									
IA	0.126	0.823	.415	0.035	0.245	.807	0.031	0.227	.822
H/I	0.207	1.307	.198	0.179	1.231	.225	0.170	1.215	.231
EPQ-P	0.040	0.261	.795	0.118	0.821	.416	0.115	0.832	.410
EPQ-E	−0.107	−0.710	.481	−0.153	−1.110	.273	−0.182	−1.372	.177
Orienting	−0.025	−0.163	.871	0.122	0.837	.407	0.072	0.507	.615
Conflict	−0.042	−0.272	.787	0.117	0.789	.434	0.067	0.465	.644
Alerting				0.305	2.217	.032	0.346	2.599	.013
EPQ-N				−0.374	−2.589	.013	−0.329	−2.352	.023
Alerting × EPQ-N							−0.296	−2.222	.032
F	0.607			1.720			2.153		
*P*	.768			.106			.036		
Adjusted R^2^	−0.062			0.118			0.190		

Alerting×EPQ-N *=* interaction between alerting effect and neuroticism, EPQ-E *=* Eysenck personality questionnaires-extraversion, EPQ-N *=* Eysenck personality questionnaires-neuroticism, EPQ-P *=* Eysenck personality questionnaires-psychoticism, H/I = Hyperactivity/impulsivity, IA *=* inattention, SNAP-IV *=* Swanson, Nolan, and Pelham, Version IV Scale.

**Table 5:** Moderators of cognitive flexibility perseverative errors (PRE).

	Model I			Model II			Model III		
	β	t	*P*	β	t	*P*	β	t	*P*
Sex	0.173	1.055	.297	0.156	1.043	.303	0.103	0.731	.469
Age	−0.137	−0.879	.384	−0.179	−1.256	.216	−0.136	−1.019	.314
SNAP-IV									
IA	0.159	1.042	.303	0.068	0.480	.634	0.063	0.478	.635
HI	0.205	1.298	.201	0.178	1.237	.223	0.167	1.241	.221
EPQ-P	0.051	0.332	.741	0.127	0.891	.378	0.123	0.927	.359
EPQ-E	−0.076	−0.508	.614	−0.123	−0.903	.371	−0.158	−1.239	.222
Orienting	−0.094	−0.630	.532	0.051	0.354	.725	−0.010	−0.070	.945
Conflict	−0.105	−0.687	.495	0.057	0.388	.700	−0.003	−0.025	.980
Alerting				0.320	2.351	.023	0.369	2.886	.006
EPQ-N				−0.367	−2.569	.014	−0.313	−2.326	.025
Alerting × EPQ-N							−0.357	−2.786	.008
F	0.675			1.841			2.637		
*P*	.711			.081			.011		
Adjusted R^2^	−0.051			0.135			0.250		

Alerting×EPQ-N *=* interaction between alerting effect and neuroticism, EPQ-E *=* Eysenck personality questionnaires-extraversion, EPQ-N *=* Eysenck personality questionnaires-neuroticism, EPQ-P *=* Eysenck personality questionnaires-psychoticism, H/I = hyperactivity/impulsivity, IA *=* inattention, SNAP-IV *=* Swanson, Nolan, and Pelham, Version IV Scale.

**Table 6:** Moderators of cognitive flexibility number of categories completed (CC).

	Model I			Model II			Model III		
	β	t	*P*	β	t	*P*	β	t	*P*
Sex	−0.322	−2.066	.044	−.309	−2.167	.036	−0.285	−1.991	.053
Age	0.157	1.064	.293	0.200	1.466	.150	0.180	1.322	.193
SNAP-IV									
IA	0.080	0.551	.585	0.160	1.188	.241	0.162	1.211	.232
H/I	−0.304	−2.033	.048	−0.285	−2.071	.044	−0.280	−2.044	.047
EPQ-P	−0.046	−.316	.754	−0.108	−0.793	.432	−0.106	−.784	.437
EPQ-E	0.022	0.153	.879	0.066	0.507	.614	0.082	0.631	.532
Orienting	0.115	0.810	.422	−0.011	−0.077	.939	0.017	0.123	.903
Conflict	−0.007	−0.048	.962	−0.159	−1.138	.261	−0.131	−0.935	.355
Alerting				−0.327	−2.515	.016	−0.349	−2.678	.010
EPQ-N				0.306	2.240	.030	0.281	2.049	.047
Alerting × EPQ-N							0.163	1.247	.219
F	1.399			2.449			2.396		
*P*	.222			.020			.020		
Adjusted R^2^	0.056			0.212			0.221		

Alerting×EPQ-N *=* interaction between alerting effect and neuroticism, EPQ-E *=* Eysenck personality questionnaires-extraversion, EPQ-N *=* Eysenck personality questionnaires-neuroticism, EPQ-P *=* Eysenck personality questionnaires-psychoticism, H/I = hyperactivity/impulsivity, IA *=* inattention, SNAP-IV *=* Swanson, Nolan, and Pelham, Version IV Scale.

To further explore the moderating effect, according to the procedures described by Aiken, West, Reno,^[55]^ we replaced the variable of the neuroticism in the regression model with the values of −1 SD (low EPQ-N) and + 1 SD (high EPQ-N) to examine the simple effects.

**Figure 2.**
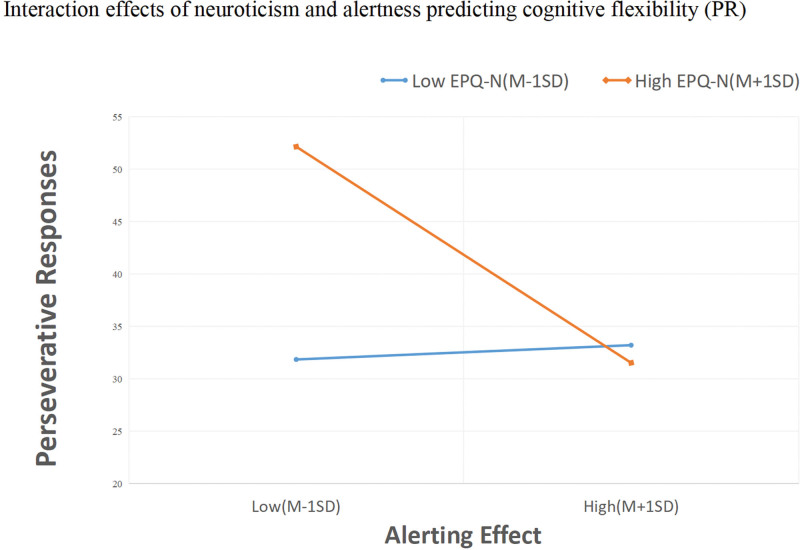
Interaction effects of neuroticism and alertness predicting perseverative responses. EPQ-N = Eysenck personality questionnaires-neuroticism, M = mean; SD = standard deviation.

**Figure 3.**
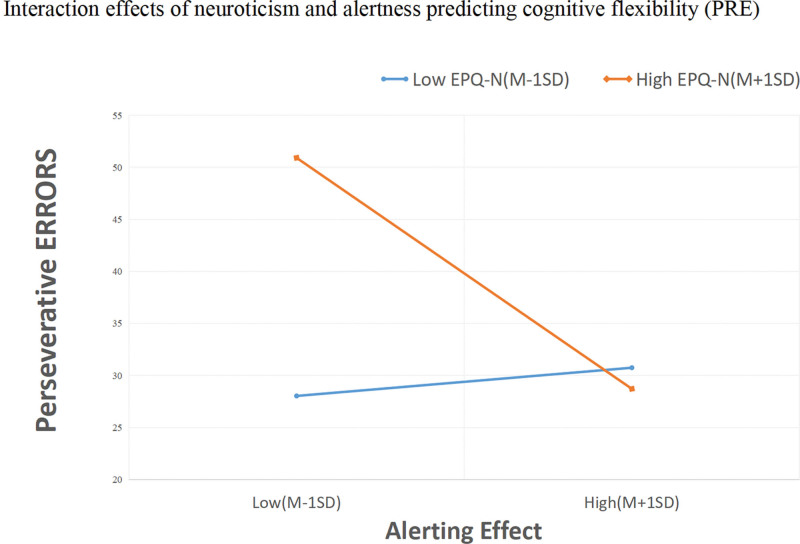
Interaction effects of neuroticism and alertness predicting perseverative errors. EPQ-N = Eysenck personality questionnaires-neuroticism, M = mean, SD = standard deviation.

Figures 2 and 3 showed the simple effects, and simple slope analysis revealed that the alerting impact was associated with PR (b = −0.28, t = −3.75, *P* < .0001) and PRE (b = −0.30, t = −4.28, *P* < .0001) at high neuroticism levels. As the alerting efficacy of children with ADHD declined, they demonstrated increasing errors in cognitive flexibility, indicating worse cognitive flexibility. However, this association with PR (b = 0.018, t = 0.219, *P* = .827) and PRE (PRE, b = 0.08, t = 0.46, *P* = .65) was not significant for low neuroticism.

## 4. Discussion

Based on our literature review, the main findings indicated that children with ADHD had significantly higher PR and PRE on the WCST and a statistically lower CC than the control group. At high levels of neuroticism, the alerting effect was strongly associated with cognitive flexibility, and this association was not salient in participants with low levels of neuroticism.

As mentioned previously, we observed that the number of errors in cognitive flexibility was significantly higher in the ADHD group than in the control group, which is consistent with our hypothesis, implying a deficit in cognitive flexibility in ADHD. This finding is consistent with previous studies findings.^[10,15,16,18,56]^ The findings of Rommelse, Altink, de Sonneville, Buschgens, Buitelaar, Oosterlaan, Sergeant^[19]^ are inconsistent with ours, which indicated that children with ADHD and their unaffected siblings did not present evidence of impairments in cognitive flexibility. This finding may be because Rommelse, Altink, de Sonneville, Buschgens, Buitelaar, Oosterlaan, Sergeant^[19]^ used a more straightforward experimental paradigm in their study, where the rules for the participants were known and constant throughout, rather than a complex experimental paradigm such as the WCST, which has unknown and constantly changing rules.^[10]^ Moreover, the author^[19]^ considers that the deficits in EF observed in ADHD are probably due to deficiencies in lower-order cognitive functions, including attention, and do not directly influence higher-order functions, such as cognitive flexibility. A possible explanation for these inconsistent findings is that studies on children with ADHD tend to involve those across a wide age range. Moreover, the role of the accelerated development of cognitive flexibility in early school age may be obscured by the inclusion of older children.^[18,57]^

This study used a child-specific version of the ANT to measure attention network. Significant impairments in the alerting network in the ADHD group compared to the control group were observed in this study; however, no significant impairments were found in the orienting and executive networks, which is partially consistent with the findings of previous studies.^[24,58]^ Previous studies have shown differences between alertness and control networks.^[23,59]^ Such inconsistencies may be related to different ANT procedures, analysis methods, and patients inclusion criteria. In addition, we observed that the alerting effect was a predictor of cognitive flexibility after controlling for other correlates in the regression model. Alerting effect and PR or PRE were significantly and negatively correlated at high levels of neuroticism. Since a higher PR or PRE represented lower cognitive flexibility, a lower alerting effect was associated with lower levels of cognitive flexibility. This result may imply that children with ADHD have some deficits in their ability to be alert to incoming stimuli and maintain arousal to receive incoming information while processing the attentional system. It has been proposed that the primary cause of abnormal cognitive flexibility lies in attention deficits, which result in a lack of acquiring the right and consecutively to problems with processing, memorizing, and recalling information.^[22]^ Roshani, Piri, Malek, Michel, Vafaee^[15]^ argued that cognitive flexibility depends on controlling attention processes to check whether the situation has changed and whether a non-routine response is necessary. Thus, we believe that attention deficits in children with ADHD, with more appropriately impaired alertness, make them less sensitive to external stimuli and incoming information. They have challenges maintaining an alert state in response to subsequent changes, leading to abnormal cognitive flexibility. Therefore, clinicians need to provide timely emotional regulation and psychological assistance to help people with ADHD to stabilize and calm them. This measure will help improve attentional functioning and cognitive flexibility in ADHD and halt its progression.

This present study showed that children with ADHD had higher neuroticism scores than those in the control group.^[33,60]^ Contrary to our hypothesis, we found that neuroticism predicted cognitive flexibility after controlling for other correlates in the regression model. Notably, neuroticism moderating effect on the relationship between alerting and cognitive flexibility. We observed that at high levels of neuroticism, the alerting effect and PR/PRE were significantly negatively correlated, indicating that in high neuroticism ADHD, children with lower alertness had a higher PR and PRE (poorer cognitive flexibility). The moderating effect of neuroticism was not strong but significant in our analysis. Combining the results of multiple regression analysis with simple slope analyses, we deem that alerting deficit was presumably more likely to induce decreased cognitive flexibility in ADHD with high neuroticism. In other words, the failure to separate from the negative thoughts and feelings keeps ADHD with high neuroticism from connecting with the present moment,^[43]^ making it difficult to perceive whether a situation has changed, needs to be changed, or the requirement to respond accordingly.^[15]^

Neuroticism is a personality trait characteristic of a tendency to experience negative emotions more frequently, intensely, more readily, and for more extended periods. One condition that individuals often confront is the necessity to dynamically adjust their actions and thoughts to respond to the changing demands of the environment. This means that individuals must maintain their purposes and goals over time while being flexible enough to shift from one thought or behavior to another when significant changes occur.^[37]^ One possible explanation of moderating effect is that individuals with high levels of neuroticism are emotionally unstable, have difficulty in self-emotional regulation,^[40]^ and have challenges controlling their attentional processes.^[61]^ Research in normal populations suggests that neuroticism, emotional disorders,^[38]^ and emotion dysregulation^[39–41]^ are related. People with high neuroticism scores were less willing to modify their behavior in response to feedback.^[42]^ Kashdan et al^[43]^ suggest that people high in neuroticism experience excessive negative emotions, are tightly wrapped up in negative thoughts and behaviors, and have difficulty tolerating them, compromising their cognitive flexibility. In counterpoint, positive emotion states facilitate increased cognitive flexibility.^[43]^

From a neuropsychological perspective, maintaining positive affect, presumably via mild increases in neurotransmitter dopamine activity in prefrontal brain areas^[37,62]^ or the anterior cingulate cortex,^[63]^ improved cognitive flexibility. This is the primary focus of the ADHD etiology hypothesis. Methylphenidate, which enhances dopaminergic activity, is a common medication used to improve symptom management in ADHD. Research has indicated that appropriate doses of methylphenidate can enhance cognitive flexibility in individuals with ADHD.^[13]^ Rubia, Halari, Mohammad, Taylor, Brammer^[64]^ found that neural activation in many brain regions, including prefrontal areas and anterior cingulate cortex, was reduced in ADHD participants taking a placebo. However, ADHD participants taking methylphenidate exhibited activation levels comparable to controls, suggesting that methylphenidate treatment may normalize brain activations during cognitive tasks.^[64]^ The moderating role of neuroticism may be related to the activation of the dopamine system and some brain regions. The causal relationship of the effects of neuroticism on the association between alerting and cognitive flexibility requires further clarification by prospective studies. From a clinical perspective, psychiatrists should consider personality factors in the comprehensive assessment of cognitive flexibility in ADHD. Especially in individuals with high levels of neuroticism, psychiatrists should provide early psychological counseling and social support to remedy corresponding personality deficits and guide them to stabilize their emotions. In addition, whether personality factors are clinically relevant to the assessment and treatment of the ADHD population is a topical issue.^[33]^ Based on our findings, it would be wise for psychiatrists to consider personality traits when assessing a patient ability for cognitive flexibility.

The current study has several limitations. First, the cross-sectional design precludes the inference of causality. Moreover, given the limited sample size included in this study, our analysis might not be sufficient to test the entire model. Therefore, further validation using more prospective studies with larger samples is needed in future research to strengthen our findings and conclusions. Second, the self-reported nature of this study has inherent limitations, including personality traits. However, the data were collected using an anonymous online questionnaire, which may have mitigated these implications. The Eysenck personality model, one of the most used models in the study of ADHD, has been widely used. The present study used the Chinese version of the EPQ to measure ADHD personality traits, which has been applied in China for many years and has excellent reliability and validity. However, many ADHD studies now prefer to use the 5-factor personality model.^[60]^ Although an adult version of the Big Five Questionnaire is currently popular in China, the children versions have not been adopted. Hopefully, future research will continue to explore the implications of the 5-factor personality model on cognitive flexibility in ADHD once the children version of the Big Five Questionnaire is officially used in China.

This study has several practical clinical implications. First, our findings confirmed that children with ADHD had lower alertness efficacy, higher neuroticism scores, and impaired cognitive flexibility than the controls. In children with ADHD and high neuroticism, alerting effect and cognitive flexibility were significantly correlated. This finding deepens our understanding of ADHD and offers novel insights into the mechanism underlying cognitive flexibility deficits in individuals with ADHD. Based on these research findings, healthcare professionals can contemplate implementing targeted intervention measures that address various contributing factors, emphasizing the enhancement of cognitive flexibility. In cases where ADHD patients exhibit distinct neurotic features, doctors can devise individual-specific intervention plans, taking into account unique differences among patients. By enhancing the cognitive flexibility of individuals with ADHD, it is anticipated that their behavioral performance and learning abilities will improve, leading to advancements in their daily social interactions. The influence of attentional function, specifically alertness and personality traits, should also be considered in subsequent assessments and measurements of cognitive flexibility. Furthermore, knowledge of the cognitive flexibility profile, attentional function, and personality traits of children with ADHD may help to identify protective factors for cognitive flexibility.

## Acknowledgments

We want to thank Professor Fang and Wang of Ningxia Medical University for their guidance and great support. Special thanks to all the students who participated in the study, and to Dr Wu for her support and encouragement.

## Author contributions

**Conceptualization:** Xiang Zhang, Jianqun Fang.

**Data curation:** Xiang Zhang, Shaoxia Wang, Qianyun Liu, Yunyun Du.

**Formal analysis:** Xiang Zhang, Shaoxia Wang, Qianyun Liu, Chujun Wu, Yunyun Du.

**Investigation:** Xiang Zhang, Jianqun Fang.

**Methodology:** Xiang Zhang, Yanrong Wang.

**Project administration:** Xiang Zhang, Qianyun Liu, Jianqun Fang.

**Resources:** Xiang Zhang, Shaoxia Wang, Qianyun Liu, Chujun Wu.

**Software:** Xiang Zhang, Shaoxia Wang.

**Supervision:** Xiang Zhang, Yunyun Du, Yanrong Wang.

**Validation:** Xiang Zhang, Shaoxia Wang, Qianyun Liu, Chujun Wu, Yunyun Du, Yanrong Wang.

**Visualization:** Xiang Zhang, Shaoxia Wang.

**Writing – original draft:** Xiang Zhang, Chujun Wu, Yanrong Wang.

**Writing – review & editing:** Xiang Zhang, Chujun Wu, Yanrong Wang, Jianqun Fang.
